# Positive youth development through taekwondo: a journey to the black belt

**DOI:** 10.3389/fpsyg.2025.1630461

**Published:** 2025-07-04

**Authors:** Yongsun Lee, Seami Lim

**Affiliations:** ^1^Department of Arts and Humanities, Korea Science Academy of KAIST, Busan, Republic of Korea; ^2^Division of Sport Science, Incheon National University, Incheon, Republic of Korea

**Keywords:** sport based youth development, life skills, Taekwondo, psychological assets, document analysis

## Abstract

**Introduction:**

This study explored the role of Taekwondo (TKD) in fostering Positive Youth Developmental (PYD) outcomes among young practitioners. Drawing on the Sport-Based Youth Development (SBYD) framework, it investigated how the black belt journey contributes to physical, social, and emotional growth.

**Methods:**

An embedded case study design was used, focusing on four TKD academies in the Southeastern United States. Data sources included student handbooks, instructor manuals, black belt essays, and recommendation letters. Thematic analysis was employed to analyze the qualitative data.

**Results:**

Findings revealed that (1) the black belt journey offered a holistic experience integrating physical skill development with life skill acquisition; (2) the belt ranking system facilitated structured goal setting and achievement; and (3) core life skills such as perseverance, integrity, and leadership were intentionally taught within the TKD curriculum across all academies.

**Discussion:**

The study suggests that TKD can serve as an effective context for promoting PYD when it includes opportunities for setting goals, receiving behavioral guidance, and learning transferable life skills. The integration of goal-oriented progression and character education appears to be key in supporting youth development through martial arts.

## 1 Introduction

Recently, numerous martial arts federations, associations, and community organizations have recognized the potential of martial arts as an educational tool to teach life skills to youth, thereby enhancing their lives (United Nations Education, Scientific and Cultural Organization, 2019). Previous research indicated that martial arts offer a context for youth to develop life skills and achieve positive developmental outcomes (Hellison, 2011; Vertonghen and Theeboom, 2013; Wright et al., 2012). A study by Vertonghen and Theeboom (2010) found significant evidence supporting the social-psychological benefits of martial arts for youth, including improved self-esteem, respect, confidence, and self-control.

Taekwondo (TKD), a Korean traditional martial art and combat sport practiced by over 30 million people through 213 Member National Associations of World Taekwondo (WT), the international federation governing the sport of Taekwondo (World Taekwondo, 2022), intentionally teaches life skills (Cook, 2001). Its curriculum is founded on five tenets: Courtesy, Integrity, Perseverance, Self-Control, and Indomitable Spirit, which practitioners are expected to follow. Within these tenets, behavior goals such as respect, goal setting, leadership, and teamwork are taught during the journey to the black belt (Cook, 2001; Lee, 2023). Taekwondoists (used as youth Taekwondo practitioners in this study) progress through various colored belts over many years of dedication and hard work (Park and Gerrard, 2013), with specific life skills embedded in the belt system. Ultimately, martial arts practice serves as a means of self-cultivation (Barczyński et al., 2009), achieved through the journey to the black belt.

The development of life skills is a core component of Sport-Based Youth Development (SBYD) programs (Gould and Carson, 2008; Hellison, 2011; Holt et al., 2017). The field of SBYD is grounded in empirical evidence showing that sports and structured physical activity can promote positive developmental assets for youth, including physical, psychological, social/emotional, and intellectual growth (Fraser-Thomas et al., 2005). This holistic, youth-centered approach fosters developmental assets that translate into personal and social skills applicable beyond sports (Holt et al., 2017).

Martial arts and combat-sport programs have been studied by several SBYD scholars to understand positive youth developmental outcomes. For example, studies conducted by Gordon et al. (2022) and Hemphill et al. (2019) identified teaching strategies for life skills development and positive youth developmental outcomes. Unlike studies focusing solely on program outcomes, these studies examined “what happened during the lessons” to better understand the process of participants’ life skills learning. Similarly, Wright and Burton (2008) and Wright et al. (2010) integrated tai chi with the Teaching Personal and Social Responsibility (TPSR) model, using it as an SBYD context where youth participants learn life skills and responsibilities through tai chi lessons.

Although several SBYD scholars have examined the strategies for the life skills development in the context of martial arts, studies on martial arts within the SBYD framework are rare, especially concerning TKD. Unlike other martial arts (e.g., tai chi) and combat sports (e.g., boxing and wrestling), the “black belt journey” is a distinctive heritage and tradition in TKD, akin to traditional Asian martial arts like Judo, Karate, and Jiujitsu. Despite its relevance, the role of the black belt journey in fostering positive youth developmental outcomes remains conceptually underexplored in current literature. Specifically, the integration of black belt life skills and the significance of the black belt journey for positive youth development remain unclear that warrants this investigation. This study aims to fill this gap, providing insights for SBYD scholars and practitioners on how TKD can facilitate positive youth developmental outcomes.

## 2 Conceptual framework

The present study focused on positive youth development in the TKD context, specifically on the experiences of youth Taekwondoists in their journey to achieving the black belt. This exploration was based on the SBYD conceptual framework, a youth-centered approach to enhancing developmental assets through sport and physical activity (Fraser-Thomas et al., 2005; Holt and Neely, 2011). The field of SBYD is grounded in the need to conceptualize the processes that promote developmental outcomes. SBYD scholars particularly aim to gain a deeper understanding of the contextual features of SBYD programs (Cote et al., 2014; Jacobs and Wright, 2018; Holt et al., 2017). Three context-related factors are notably highlighted: (1) motivational environment, (2) positive youth development (PYD) climate, and (3) program design and approach. These factors should be combined to effectively design and implement SBYD programs (Beesley and Fraser-Thomas, 2019; Holt et al., 2017).

First, the motivational environment has been found to influence positive youth PYD outcomes. This environment includes features such as positive social norms and values (Hemphill et al., 2019; Hemphill et al., 2022; Jacobs and Wright, 2018), specific goals, rules, and expectations (Hellison, 2011), and physical and psychological safety (National Research Council and Institute of Medicine (NRCIM), 2002). Within such a motivational environment, youth feel safe to take risks, challenge themselves, and learn from their mistakes (Bean et al., 2018; Danish et al., 1993). This environment is reinforced by building positive relationships, creating a PYD climate (Holt et al., 2017).

The relational factor, such as building positive relationships, has consistently been recognized as contributing to PYD outcomes. When youths are surrounded by caring adults (e.g., coaches or program leaders) and build positive relationships with their peers and these adults, PYD outcomes are more likely to occur (Petitpas et al., 2005). One SBYD-specific model that emphasizes relationship building is Hellison’s personal and social responsibility model (TPSR) (Hellison, 2011). ‘Relational time’ (p. 49) is a key component of the daily program format in SBYD programs adopting the TPSR model. When relationship-building time and activities are an integral part of daily lessons and programs—before or after the lesson, or whenever possible—it likely facilitates student learning and PYD outcomes (Hemphill et al., 2022). In a PYD climate, youth feel part of the learning community (Strachan et al., 2018), willingly work with others during partner and group activities, and open up to share more ideas and opinions (Lee, 2023).

Finally, SBYD programs that intentionally integrate life skills learning into sport and physical activities and facilitate the transfer of these skills to other areas of youth’ lives can positively influence overall PYD outcomes (Hellison, 2011; Jacobs and Wright, 2018). High-quality SBYD program design and approach provide opportunities for youths to understand what life skills are being taught (e.g., becoming aware of life skills or goals), practice these skills through sport and physical activities, and connect the skills to desired settings and situations outside the program or gym (Jacobs and Wright, 2018; Martinek and Lee, 2012; Pierce et al., 2017). To facilitate life skills development, handbooks that describe the skills, values, or program goals are utilized in several SBYD programs, such as the restorative youth sport program (Hemphill et al., 2022; Lee, 2023) and the Naenae Boxing Academy (Gordon et al., 2022; Hemphill et al., 2019). Debrief sessions, group meetings/discussions, and restorative circles provide space for life skills awareness, reflection, and cognitive connections for the transfer of life skills (Hellison, 2011; Hemphill et al., 2022; Jacobs and Wright, 2018; Pierce et al., 2017).

Although SBYD literature provides clear conceptualizations and rich information about facilitating PYD outcomes, what causes such outcomes in youth martial arts programs still needs to be explored. The founder of the martial art of Aikido, Morihei Ueshiba, stated that “The way of a warrior is based on humanity, love, and sincerity; the heart of martial valor is true bravery, wisdom, love, and friendship. Emphasis on the physical aspects of warriorship is futile, for the power of the body is always limited” (Ueshiba, 1992, p. 59). The warrior’s pathway refers to “pushing one’s own limits, a kind of transgression through continuous effort of self-development—it is a moral way, improving the character and personality of the fighter through his own weakness” (Cynarski and Lee-Barron, 2014, p. 12). The values and goals of this pathway are not much different from what youth experience in quality SBYD programs. The TKD black belt journey is the warrior’s pathway that involves the TKD system, curriculum, philosophy, culture, and tradition. Research documenting the TKD program design, curriculum, and approach to life skills development, as experienced by youth Taekwondoists in their journey to the black belt, can help advance the body of knowledge related to understanding SBYD through TKD and its outcomes. The purpose of this study was to explore positive youth development in youth Taekwondoists’ black belt journey through the lens of the conceptual framework for SBYD. The following research questions guided the data collection and analysis: (1) How does the black belt journey integrate the teaching of life skills? (2) How does the journey impact youth Taekwondoists’ lives?

## 3 Materials and methods

### 3.1 Research design

**Figure 1:**
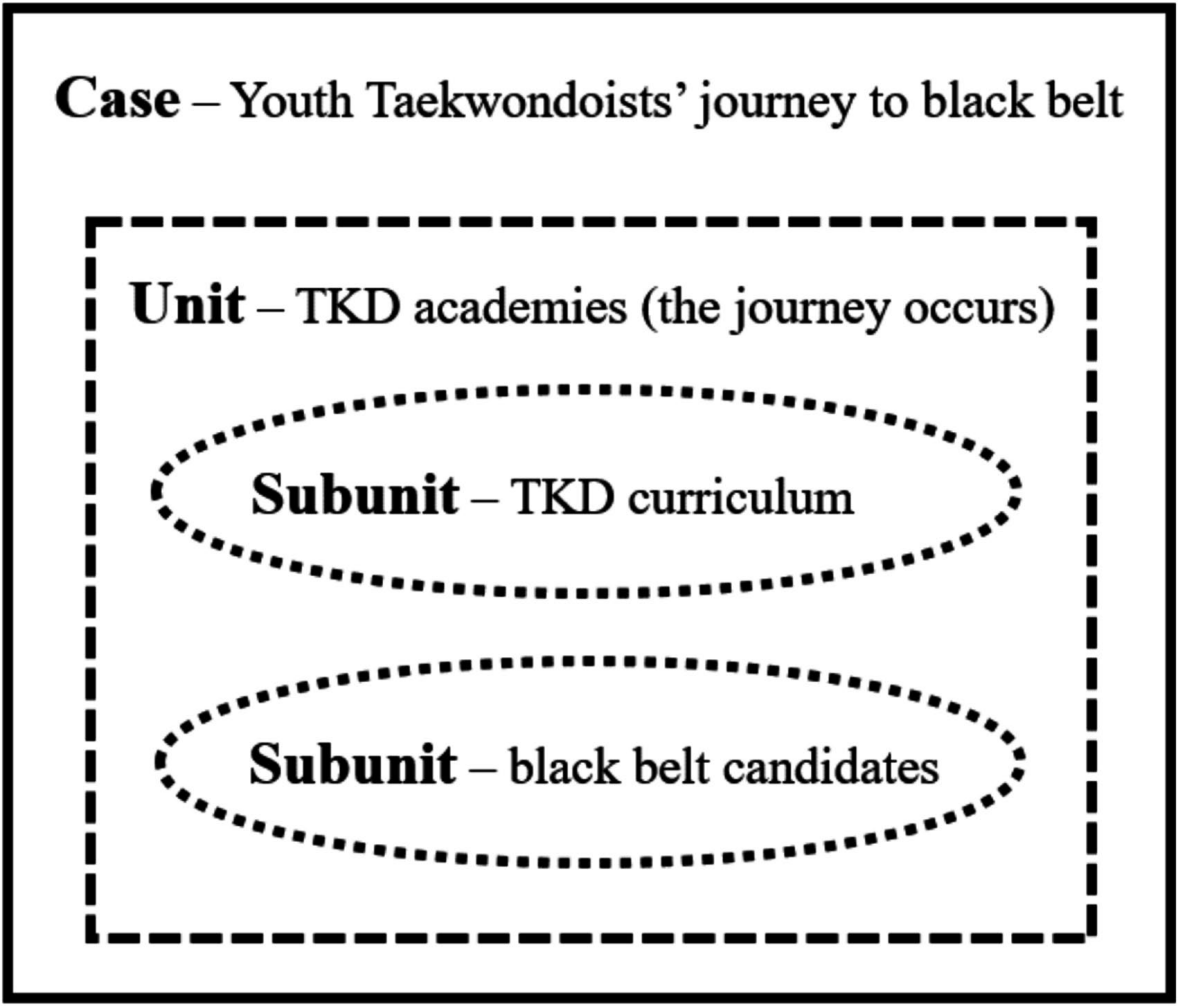
The embedded case study design with a particular case and multiple units of analysis.

The current study employed an embedded case study design to explore youth Taekwondoists’ journey to the black belt as a process of positive youth development. A case study is a research method aimed at understanding a subject, a small group, an entire program, a decision process, an activity, or a specific project (Creswell, 2014; Yin, 2018). This case study identified ‘youth Taekwondoists’ journey to the black belt’ as the specific case. A case study can involve units of analysis at more than one level, known as an embedded case study (Yin, 2018). The case of this study was the black belt journey, and it involved multiple (sub)units: TKD academies that provide the black belt program, the TKD curriculum (journey to the black belt), and black belt candidates who experience the journey. Figure 1 illustrates a visual concept of the case and its (sub)units.

### 3.2 Sampling design

Sampling is the process of selecting part of a population or universe (Gliner et al., 2017). In general, population refers to a number of people, but it can also mean total quality of the things, cases, and events (Gliner et al., 2017; Etikan et al., 2016). Purposeful sampling was used to carefully select information-rich settings that provided the most relevant data set. It allowed investigators of the present study an in-depth understanding of PYD process in youth Taekwondoists’ journey to black belt. An attempt was made to set a specific criteria to select settings: (1) life skills, character development, or values were specified and described on the websites of TKD academies in the Southeastern region of the United States that the first author of this study can make observation, collect data, and communicate with research participants, (2) TKD academies that provide a black belt program for children/youth, (3) TKD academies that have common characteristics, such as WT (World Taekwondo) style of TKD, (4) the investigators can access to rich information and divers sources of data, and (5) easy accessibility, geographical proximity, availability for further communication at a given time are available. Based on the specific criteria, four TKD academies were selected: Victory TKD, Triumph TKD, Champ TKD, and Glory TKD (pseudonym was given to the academies). The reason for choosing to select the four TKD academies was because the evidence from multiple academies was considered more compelling and robust as compared to a single unit (a TKD academy). The sample was limited to four academies because no more than four academies wanted to participate in the current study as they were asked to share sensitive information about the students or the curriculum.

### 3.3 Brief descriptions of the four TKD academies

The general information and characteristics of the four TKD academies are briefly described below, including the background of the owner/master, goals of the academies, program focus, and student characteristics. All four academies follow the World Taekwondo (WT) style, which is the internationally recognized form of South Korean Taekwondo. Table 1 presents detailed general information and characteristics of the four Taekwondo academies. The enrollment numbers, gender distribution, and racial composition were obtained directly from each academy’s administrative staff during the data collection phase and are presented as approximate estimates, reflecting the general information provided at the time.

**Table 1:** Characteristics of the four TKD academies.

Name	Victory TKD	Triumph TKD	Champ TKD	Glory TKD
Owner/master	Master K	Master J	Master L	Master S
Instructor/Staff (#)	Instructor (2)	Instructors (2)	Instructor (2)	Instructor (1)
	Manager (2)	Manager (2)	Manager (1)	Manager (1)
Student enrollment	210 students	250 students	120 students	100 students
Racial enrolment	White: 73%	White: 76%	White: 65%	White: 63%
	Hispanic: 12%	Black: 12%	Black: 17%	Black: 22%
	Black: 10%	Hispanic: 6%	Hispanic: 15%	Hispanic: 12%
	Other: 5%	Other: 6%	Other: 2%	Other: 3%
Student sex ratio	M-67% / F-33%	M-72% / F-28%	M-71% / F-29%	M-68% / F-32%
Marjory student age	K-1 – K-8	K-1 – K-8	K-1 – K-8	K-1 – K-8
Age range of youth participants	Ages 7–18	Ages 7–15	Ages 12–16	Ages 9–13

#### 3.3.1 Victory TKD

Victory TKD was founded by Master K in 1998. Master K with a 7th-degree black belt in TKD, has over 35 years of teaching experience in the U. S. Victory offers various programs, including Little Tigers TKD (ages 4–5), children/youth programs (ages 7–18), adult programs, family programs, summer camps, and after-school programs. The children/youth program features an age-specific TKD curriculum with activities for fun and fitness, as well as opportunities for life skills development (e.g., respect, self-control, social skills, and self-confidence). The program also includes anti-bullying activities. Youth Taekwondoists (ages 7–18) attend three 40-min sessions per week. At the time of data collection, approximately 210 students were enrolled at Victory, with 67% male and 33% female. The racial distribution of the students was approximately: White (73%), Hispanic (22%), Black (10%), and other (3%). Victory has two TKD instructors and two managers serving students and parents.

#### 3.3.2 Triumph TKD

Master J opened Triumph TKD in 2009 with a strong focus on the personal development of young students. Originally from South Korea, Master J has a professional background in teaching TKD and coaching national teams, including the US National Team in 1997 and 2000. He holds a 7th-degree black belt in TKD and has over 20 years of experience focusing on morals, values, and core character traits in his teaching. Triumph TKD offers various programs, including children’s programs for ages 3–12, teen and adult programs, family programs, after-school programs, and summer camps. Youth Taekwondoists (ages 7–15) attend two 50-min sessions per week. At the time of data collection, approximately 250 students were enrolled at Triumph, with 72% male and 28% female. The racial distribution was approximately: White (76%), Black (12%), Hispanic (6%), and other (6%). Triumph TKD has two TKD instructors and two managers serving students and parents.

#### 3.3.3 Champ TKD

Since 2012, Master L, head instructor at Champ TKD, began training in TKD during his school years and earned a bachelor’s degree in TKD from YONG IN University. As captain of the university’s demonstration team, he gained international experience by performing and training across Asia, America, and Africa. His global background and educational expertise inform the academy’s age-appropriate programs, which emphasize both martial arts skills and life values such as confidence, discipline, and respect. His program emphasizes that youth Taekwondoists can maximize their potential and accelerate their development through TKD training and life skills learning (e.g., focus, discipline, respect). The primary goal of the teen TKD program is to boost their confidence and help them make friends as they progress to the black belt. Youth Taekwondoists (ages 12–16) attend three 45-min sessions per week. At the time of data collection, approximately 120 students were enrolled at Champ, with 71% male and 29% female. The racial distribution of students was approximately: White (65%), Black (17%), Hispanic (15%), and other (2%). Champ TKD is served by two TKD instructors and one program manager who support the students and their parents.

#### 3.3.4 Glory TKD

The TKD programs at Glory TKD academy are based on the teaching philosophy of Master S, which maintains the traditional aspects of martial arts training (e.g., bowing, showing mutual respect, and answering “yes, sir/ma’am”). Master S holds a 7th-degree black belt in TKD and has over 25 years of teaching experience in the U. S. His program focuses on improving students’ health in body, mind, and spirit through TKD training, with the belief that a healthy body promotes a healthy mind and spirit. The TKD program targets children, teens, and adults. Youth Taekwondoists (ages 9–13) attend two 45-min sessions per week. At the time of data collection, approximately 100 students were enrolled at Glory, with 68% male and 32% female. The racial distribution of students was approximately: White (63%), Black (22%), Hispanic (12%), and other (3%). Master S was the main instructor, covering all sessions with the help of student instructors (leadership team). One program manager served the TKD students and their parents at Glory.

### 3.4 Data collection

The current case study had several units of analysis, with the TKD academies as the main units and the TKD curriculum and youth TKD black belt candidates as subunits, all embedded in the journey to the black belt. Attention was given to these units because different data could come from various sources of evidence (Yin, 2018). For this study, all types of documents (pre-existing textual data) were collected as primary data sources to understand the context, TKD curriculum, system, and youth black belt TKD candidates’ learning experiences.

First, the website content of the TKD academies was carefully reviewed to provide context regarding program goals, mission, vision, teaching philosophy, and types of programs offered. Second, physical evidence, including objects found within the study setting, can be utilized as a data source (O’Leary, 2014). TKD student handbooks, instructor manuals, junior instructor manuals, TKD curriculum manuals, and TKD belt promotion test forms were important data sources as physical evidence for this study. Moreover, black belt essays, black belt training logbooks, and letters of recommendation for black belt candidates were used to understand youths’ black belt journey. The black belt essay is a reflection on the black belt journey, including what the black belt means to them, their passion and excitement about being a black belt candidate, any personal changes since starting TKD, who helped them achieve their goal, and what led them to this achievement. The black belt training logbook outlines all requirements to be completed before the black belt test, including leadership logs, black belt life skill speeches, volunteer history, and information about required seminars and workshops. Letters of recommendation for the candidates are written by peers, family members, teachers, or friends, supporting the candidate’s positive character and eligibility for the black belt test. The number of documents collected from the four academies is shown in Table 2.

**Table 2:** Physical evidence (document) collected from the four TKD academies.

Academy	Student handbook	Instructor manual book	Curriculum manual book	Belt promotion test form	Black belt essay	Black belt logbook	Letter of recommendations
Victory	Yes	Yes	Yes	Yes	20	10	10
Triumph	Yes	Yes	Yes	Yes	20	10	10
Champ	No	No	Yes	Yes	5	5	5
Glory	No	No	Yes	Yes	8	5	5
Total	2	2	4	4	53	30	30

(1) Student handbook (student-book); (2) Instructor manual book (instructor-book); (3) Curriculum manual book (curriculum-book); (4) Belt promotion test form (testing form); (5) Black belt essay (BB essay); (6) Black belt log book (BB log); (7) Letter of recommendations (LoR).

### 3.5 Reasons for conducting document analysis

In qualitative studies, data is collected through various methods. While qualitative researchers often prefer to create data, they sometimes rely on pre-existing data depending on the research questions and available data sources (Bowen, 2009; Morgan, 2022). Stake (1995) and Yin (2018) highlight that document analysis is particularly suitable for qualitative case studies, producing rich descriptions of the case or program. Merriam and Tisdell (2016) state that pre-existing data is comparable to interviews and observations, as ‘texts’ provide information similar to these methods. Although triangulation of data from different sources enhances the validity of qualitative research, interviews and observations can be challenging to conduct in certain research circumstances (Morgan, 2022).

The first author of this study was invited to judge a black belt test. During this opportunity, several black belt candidates were asked if they could participate in the study. They responded, “Everything you want to know is in our black belt essays and black belt training logs” (personal communication, August, 2022). The essay reflects the TKD black belt journey, including what the black belt means to them, their passion and excitement about being a candidate, personal changes since starting TKD, who helped them achieve their goal, and what led to this achievement. These components were what researchers planned to explore through interviews. Thus, the essays, as pre-existing documents, provided valuable insights into their journey to the TKD black belt. The black belt essays and recommendation letters were reviewed by the masters and program staff. Samples of these documents were shared with the study authors, confirming they provided rich information comparable to interviews. The feasibility of document analysis outweighed the benefits of using other data collection methods for this study. This study was approved by the Institutional Review Board of UNGC (Approval No. IRB-FY21-214).

### 3.6 Data analysis

A thematic analysis was conducted to analyze the documents. This process involved skimming/reading through a data set and identifying repeated patterns of meaning to derive themes. The analysis followed the approach outlined by Braun and Clarke (2006), which consists of six steps: (1) familiarizing with the data, (2) creating initial codes, (3) collecting codes with supporting data, (4) grouping codes into themes, (5) reviewing and revising themes, and (6) producing the report/manuscript.

The authors used a deductive approach to pattern recognition and theme identification. The authors started by developing a rough codebook with an initial set of codes based on the existing SBYD framework (e.g., conceptualization for PYD facilitation and best practices for life skills learning). A set of predetermined codes allowed researchers to find excerpts from documents that fit those codes. As coding progressed, the codebook evolved, with new codes added and categories reorganized.

To enhance credibility and validity, data triangulation, peer debriefing, and member checking were employed (Lincoln and Guba, 1985). Multiple documents were collected from different samples (research sites). Black belt essays, training logbooks, and recommendation letters from the four TKD academies were triangulated to compare evidence and gain deeper understanding. Peer debriefing involved a second author proficient in qualitative research procedures with an impartial view to assess methodology and findings (Anney, 2014; Hail et al., 2011). Finally, member checking was carried out by maintaining regular contact with the four TKD masters throughout the data collection period to discuss categories and themes from the analysis (Carlson, 2010; Curtin and Fossey, 2007).

## 4 Results

### 4.1 TKD black belt: more than just kicks and punches

Two overarching categories characterized the first main theme, “TKD black belt: More than just kicks and punches.” The two categories were (1) meaning of TKD and (2) meaning of the black belt journey. The first category related to Taekwondoists’ general perception of TKD and what it means to them. The second theme was about their reflection on their black belt journey. The primary data sources generating these categories included BB essay and LoR.

#### 4.1.1 Meaning of TKD

The findings were grouped based on Taekwondoists’ learning experiences (more than three years) in their TKD academies. Three codes were identified within this category: Out of comfort zone, Part of my life, and Applied strength. Most black belt candidates described their desire to change and improve by engaging in new activities and unique experiences. The practice of Taekwondo inspired them to step out of their comfort zones, and they generally accepted that personal growth happened outside of these zones. They leveraged it to develop themselves through the practice of TKD. One female candidate shared, “TKD has pushed me out of my comfort zone numerous times, into a loud, active environment that I would not usually interact with” (BB essay4-youth). Another female candidate noted: “I was very shy; I did not make many friends before. TKD helped me get out of the fog. I got better at talking to people and working with others” (BB essay23-youth).

Many Taekwondoists perceived TKD as an important part of their lives, beyond just learning physical skills. To qualify as black belt candidates, Taekwondoists had to practice for at least 3 years and were expected to train regularly. A parent of a youth candidate described, “We attended class 3–4 days a week like clockwork” (BB essay1-Family). All candidates took TKD seriously, considering it a primary part of their lives. A co-director of an afterschool program highlighted in her letter of recommendation the strong commitment to TKD practice:

In the year that I’ve worked with Shayla, my team and I have seen just how committed she is to TKD. Some days she misses performing arts club to make sure she is in attendance for TKD … Shayla also speaks on working on her TKD expertise on the weekends and over the summer. It is truly wonderful to see her overjoyed by this incredible program (LoR13-Director).

TKD was not only an essential part of their lives due to their dedication, but also because it provided significant lifelong benefits. A male candidate shared, “TKD has always been a big part of my life. It has taught me many things that have not only helped me in TKD, but in all areas of my life” (BBE26-Youth). Similarly, a female candidate stated, “TKD has helped me in many aspects of my life. It has helped me gain confidence, cooperate with those around me, and take on more responsibility” (BB essay8-youth). These findings indicated that TKD was integral to their lives because they recognized its value and benefits.

For candidates, TKD offered a unique experience that involved various ways to move their bodies. Many believed that TKD practice required a variety of movements distinct from other sports and physical activities, yet applicable to them. One candidate referred to this as “applied strength or power” in her black belt essay:

I knew that working my muscles in a new way (differently from my time at the gym) would physically make me stronger. I was already taking care of myself pretty well physically, so this new feeling of strength was unexpected. Maybe the best wording is applied strength, or power. There is something powerful feeling about all facets of TKD, whether it’s kicking a body shield or breaking a board (BBE2-Family).

A male candidate described his perspective on applied strength, explaining how TKD can be utilized outside the TKD academy. He stated:

I can use TKD to help me and others in scary situations. If I ever fall down the stairs, I would remember what I was taught to roll my body so I won’t get hurt. If someone is trying to attack me, I would use self-defense techniques I learned to protect myself. If anyone tries to hurt my family or friends, I would use my defense moves to protect them and make sure they are safe (BB essay9-youth).

The meaning of TKD to the candidates were not fully captured by our findings alone. However, through data review and pattern identification, three key themes emerged: out of comfort zone, part of my life, and applied strength. In addition to the meaning of TKD, our findings extended to the meaning of the black belt journey, which was characterized by three codes: (1) well-rounded person, (2) new challenges, and (3) commitment.

#### 4.1.2 Meaning of black belt journey

##### 4.1.2.1 Well-rounded person

The black belt candidates in this study predominantly believed that the journey was valuable for cultivating the whole person. Many youth candidates reflected on this in their essays, stating, “To continue my journey as a black belt is to continue to grow into a better athlete and person” and “TKD is helping me grow into a diverse, well-rounded person.” Throughout the journey to black belt, students experienced personal improvement and development. For instance, a female candidate said, “I’m really glad that I got to progress this far in doing TKD, and the experience was amazing. I love doing TKD and I want to become better and better as I go on” (BB essay8-youth).

##### 4.1.2.2 New challenges

As candidates generally believed in self-improvement through their black belt journey, they considered the new challenges they faced as integral to the experience. To them, the journey to black belt involved numerous challenges that required them to demonstrate improvement in TKD skills and positive attitudes. For many parents, supporting their children in the black belt journey was motivated by the belief that their children could be mentally, emotionally, and physically challenged by the new tasks and skills required for advancement in the belt ranking system. One parent shared, “I believe she faces new challenges and continues to grow in TKD” (LoR26-parent). A female candidate reflected, “I like to be challenged and getting a black belt was a long journey with many challenges along the way” (BB essay32-youth). For many, the journey to black belt was synonymous with facing new challenges. However, passing the black belt test did not signify the end of this journey; rather, it means continually encountering new challenges. A boy candidate described these ongoing challenges in his essay:

The excitement of earning my first black belt was beyond words. I felt like it was the best thing I had ever accomplished. There was only one mistake, I thought the hard part was over. Little did I know, a new challenge was about to begin. But I enjoyed every step along the way. Here I am getting ready to do it again (BBE23_Youth)

According to a grand master at Triumph TKD academy, “Getting a black belt is not the end of your journey-it’s a new beginning and new challenges” (Research note, 11/12/2022). During black belt testing, it is emphasized that more challenging moves, forms, and sparring await beyond the first degree in black belt. Our findings suggested that continuing the journey and facing new challenges was an opportunity to start anew with better knowledge and experience in TKD.

##### 4.1.2.3 Commitment

Another significant theme identified in this study regarding the black belt journey was commitment. A parent perceived the journey as a commitment, stating, “This is a commitment, and our goal needs to be attained—our black belts” (BBE7_Family). A female candidate echoed, “A journey to becoming a black belt is not easy; it takes hard work and commitment” (BB essay32-youth). Black belt candidates needed to commit to attending classes regularly and putting forth their best effort to earn their belts and progress on time. Despite occasional reluctance to attend classes, the candidates persevered and ultimately succeeded. A parent reflected, “Our journey started down a path that took many twists and turns, but I am glad that we did it” (BBE7_Family), highlighting that the black belt journey was all about commitment.

### 4.2 The roadmap for the black belt

The findings were grouped according to the roadmap for the black belt identified across the curricula of the four TKD academies and the learning experiences of the black belt candidates. As results, two categories represented the second theme. These categories were: (1) belt ranking system as stepping stones and (2) essential element of TKD: black belt life skill. The primary data sources supporting this theme included instructor books, curriculum books, testing forms, black belt essays, black belt logs, and letters of recommendation.

#### 4.2.1 Belt raking system as stepping stones

**Figure 2:**
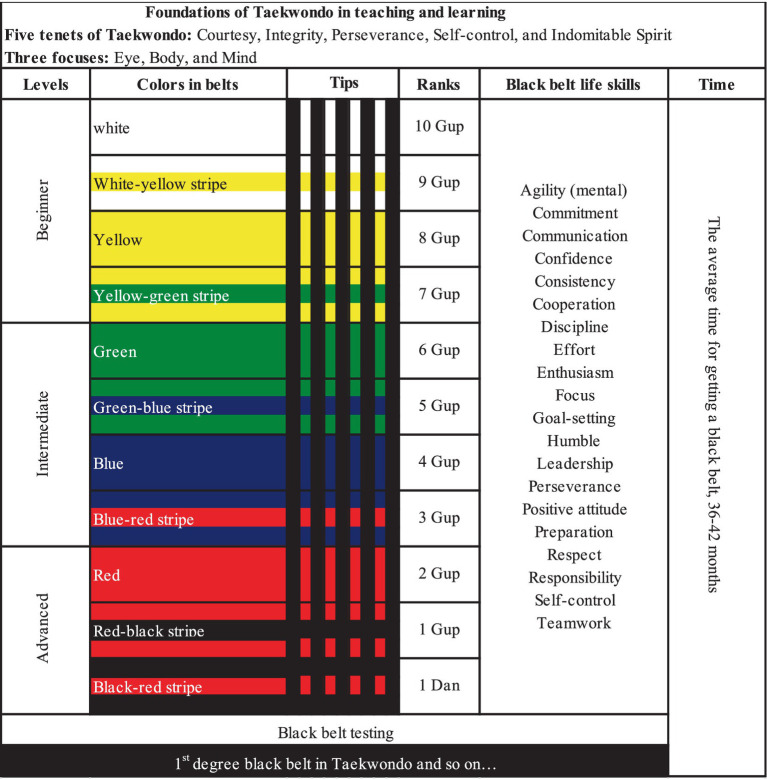
Belt ranking system with associated life skills.

To qualify for a black belt, all Taekwondoists required to pass a specified belt promotion test, which was organized every two, three, or up to 6 months, depending on the TKD academy. All TKD academies have a belt ranking system with strict promotional criteria. The system uses colored belts to represent ranks, with specific colors varying between organizations (International TKD Federation and World TKD) and from academy to academy. Despite some variations, there is a lot of similarity in the belt systems used across organizations and academies. The four cases we examined follow the World TKD style, recognized as South Korean TKD. Findings from these cases showed a similar belt system, including white, yellow, green, blue, red, black, and colored stripes between each color. Although some TKD academies included additional colors, such as purple, orange, and brown, they commonly used the five primary colors with colored stripes in their ranking system. Figure 2 shows the belt ranking system identified in this study.

In the black belt journey, we identified three levels across the four TKD academies: beginner, intermediate, and advanced. Each level included several ranks represented by different colored belts. The beginner level included white, white-yellow, yellow, and yellow-green belts. The intermediate level comprised green, green-blue, blue, and blue-red belts. The advanced level consisted of red, red-black, and black-red belts. At each rank, Taekwondoists were required to earn five tips or stripes by consistently demonstrating competence in the given learning materials (skills and tasks). According to curriculum books and testing forms analyzed in this study, the tips system tracked the achievement of short-term goals to enhance the learning experience and ensure that all Taekwondoists were well prepared for advancement to the next belt level. In this system, there is a stripe for each skill to be learned for each belt rank, representing mini-steps between the ranks, similar to a check-up or quiz. The four TKD academies used the same tips system, except for the colors of the stripes. Two academies used black stripes for tips, while the other two used white, yellow, green, blue, and red stripes.

Through document analysis, we found that the TKD belt ranking system played a crucial role as the foundation of the black belt journey. Many candidates reflected on the importance of the belt system in their journey to the black belt. For example, a male candidate elaborated, “Having a real marker in my progress, in terms of belts, has most definitely helped me feel like I am marking progress in at least one part of my life” (BB essay4-youth). He highlighted that his achievements in TKD made him feel like he was making progress in his life. Candidates vividly remembered their feelings when they earned tips or new belts, which highly motivated them to move forward. Another male candidate explained:

As a white belt when I earned my first tip, I was excited that I had accomplished something new. When I earned my second belt I was motivated and I felt like I could move forward and keep earning new belts. Now I am a poom belt. I will be testing soon to earn my black belt (BB essay13-Youth)

The TKD belt ranking system provides opportunities for Taekwondoists to set short-term goals (e.g., tips and colored belts) and long-term goals (e.g., black belt). This system allowed them to clearly identify the skills they need to practice. It also helped instructors ensure that their students were well-prepared for advancement to the next belt level, learning new skills. In the belt ranking system, forms or patterns (called poomsae), sparring, breaking, and self-defense were systematically organized and taught as essential components of the TKD curriculum across the four TKD academies. The implementation of the TKD curriculum in these academies not only focused on teaching hard skills (e.g., TKD techniques) but also addressed soft skills (e.g., respect, self-control, and leadership) according to their curriculum book and testing forms. These soft skills were systematically organized as “black belt life skills” and specified for each belt rank, requiring students to demonstrate knowledge of these particular skills.

#### 4.2.2 Essential element of TKD: black belt life skills

Analysis of documents across the four TKD academies indicated that teaching black belt life skills was equally important in the journey to achieving a TKD black belt. The TKD curriculum focused on both hard skills and soft skills. At each rank, a specific black belt life skill is taught during TKD class. Taekwondoists needed to demonstrate a satisfactory level of knowledge about black belt life skills and behaviors associated with these skills. The importance of learning black belt life skills was detailed in documents such as testing forms and curriculum books. A testing form used in Triumph TKD academy showed that:

As you are aware, the physical development of our students is only a small part of what our TKD academy offers its students. In addition to physical skills, we put a heavy emphasis on the development of character, confidence, and focus. Not only do we enjoy watching our students grow within the dojang (a training hall), but we also love to hear how they are doing outside of classes while at home and school. With this in mind, we are asking for your feedback. We have assigned a Black Belt Life Skill to each belt level. You (yourself or as a parent) have the option to complete these forms and to share your thoughts and observations. (Testing form-Triumph)

The testing forms were handed out to students or parents before their belt promotion tests. Throughout the journey to TKD black belt, Taekwondoists had opportunities to learn a wide range of black belt life skills (see Figure 2). For example, if the assigned life skill is honesty, the testing form outlined specific behaviors such as “does not cover up their mistakes.” Another example was when teaching courage, where the behavior guidelines involved “willingness to try new things” and “curiosity and inquisitiveness - asks questions” (Testing form - Triumph). Table 3 illustrates a few selected black belt life skills (first column) with expected behaviors (second column) from belt promotion testing forms, and the experiences of Taekwondoists or observations from parents or school teachers linking to the particular black belt life skills (third column).

**Table 3:** Black belt life skills and participants’ elaborations.

Examples of black belt life skills	Expected behaviors (selected a few)	Participant’ elaborations
Integrity (from Triumph)	Tries to incorporate Black Belt Life Skills into daily life Take responsibility for own actions	TKD has helped me be responsible in my life as well. You must always try your best even when people are not watching (BB essay26-youth)
Confidence (from Victory)	Unafraid to ask for help or offer help to others Speak up when they have an idea or suggestion	Jason offers his assistance to teachers and his peers, seeks to help those in need, puts others before himself, and is very hard working (LoR16byTEACHER)
Cooperation (from Champ)	Willing to admit mistake Accept diversity Show self-control when practicing with others	Joining leadership team has taught me a lot about elements of TKD. Helping kids and working with other people has helped me improve my social skills greatly (BB essay4-youth)
Patience (from Glory)	Realize that goal-setting is important and it takes time/effort to reach goals Understand that form is important (strives to keep proper technique during class)	The lessons he learned over the past 4 years will stay with him for his life. He will be able to set clear objectives and goals for himself and then have the discipline, confidence, resilience and perseverance to achieve them (BB essay-parent)

The integration of black belt life skills was an essential part of TKD curriculum. The evidence was clearly shown in documents reviewed for this study. Taekwondoists, parents, and teachers elaborated the learning experience of black belt life skills related to practicing poomsae and sparring that youth Taekwondoists must prove satisfactory level of competence and mastery. The finding related to Taekwondoists’ learning black belt life skills experience showed that they associated the black belt life skill learning experience with poomsae and sparring. Poomsae refers to a TKD patterns include a sequence of TKD techniques linked together into a pattern of moves. Many candidates shared about their experience in practicing poomsae in their black belt journey that were challenging Taekwondoists and valuable for their life. A girl candidate reflected, “I like being able to learn new poomsae with different techniques although it’s not easy to memorize all the steps (BB essay15-youth).” There was basically one poomsae assigned for Taekwondoidsts to learn per belt in preparation for their next belt promotion test. Each poomsae includes 18–24 moves with combinations of hand blocks, stances, punch/kick attack. This required cognitive abilities (memorize) and focus of Taekwondoists. A parent of a youth candidate shared that “as the forms became more complex and required more discipline to learn, Jade would become impatient and frustrated but the masters would always help him to breath and refocus his energy” (BB essay1-Family). Furthermore, another parent elaborated on the value of learning poomsae as regards to black belt life skills learning, “his commitment to learning the poomsae has helped him apply this is school. His grades have improved” (LoR11byPARENT). We identified the practice of poomsae provided opportunities to learn and apply black belt life skills, such as focus, perseverance, self-control, and commitment.

In addition to poomsae, Taekwondoists learned black belt life skills from sparring. TKD sparring was taught as a means of teaching students to use the techniques they learn within their practice. Sparring was done under strict supervision of the instructor in a safe, controlled and respectful environment. Sparring was testing their TKD techniques against another person. The benefits of sparring were described by many in the documents. For example, a girl candidate mentioned that “I do not like doing sparring because I have to focus really hard to know/read what my opponent’s next kick is” (BB essay15-youth). Her experience of sparring could be related to cognitive process that focus on what the opponent tries to do and think to how to defend and earn points (what strategies I have) like playing chess. Sparring is mentally and physically demanding. Even one might expect what the opponent would do, it’s still unknown. This may cause a feeling of fear of the unexpected attacks, injuries, or pain. A girl candidate elaborated about this experience that was valuable to her. She shared:

Within TKD I have developed in many ways. One of the most noticeable is sparring, which I did not participate in before going to TKD. I never would have participated in a contact sport such as sparring before joining TKD. Being forced to react so fast to someone else attacking me is a situation I never wanted to be in, even in a controlled environment. Learning how to look at sparring as a challenge rather than a fight has helped me overcome my fear surrounding it (BB essay4-youth).

She sees sparring as a challenge not just a fighting. A parent of a girl candidate also described her perspective on the sparring as an opportunity to improve self.

Sarah has started to use strategy to help her sparring. In past testing reflections, she has always mentioned sparring as an area that she needs extra help with. It is good to see her start to overcome her fears of hurting her opponent, making multiple moves in a sequence, scoring points, and enjoying herself during the task (LoR26-1byPARENT).

Although sparring was a big challenge to Taekwondoists, they considered it as an important task that was able to learn black belt life skills. In this study, Taekwondoists learning black belt life skills from sparring was related to indomitable spirit which was one of five TKD tenets. Having indomitable spirit in sparring was found as fighting their fears, not giving up on what they fear.

Overall, the second main theme, the roadmap for the black belt indicated that the belt system incorporated into TKD curriculum systematically and directly addressed black belt life skills as essentials for successful journey to the black belt. We found that addressing the black belt life skills per colored belt helped Taekwondoists focus on both soft and hard skills and understand what true black belt means, not just physically skilled person, but also a well-rounded person knowing the values of life skills and how to carry it out. In the belt system, Taekwondoists also required to demonstrate performing satisfactory level of poomsae and sparring.

## 5 Discussion

### 5.1 The true meaning of TKD black belt

Youth involvement in martial arts has been a contentious issue in the literature on youth sport development (Lafuente et al., 2021). Educators express concerns that using a “human target” to enhance fighting skills in martial arts (Cynarski and Lee-Barron, 2014) might increase aggressive behavior among youth participants. However, our findings demonstrate that practicing kicks and punches in TKD serves as a vehicle for developing life skills, conceptualized as black belt life skills in TKD. The TKD journey to a black belt provides opportunities to develop self-confidence, step out of one’s comfort zone, and tackle new challenges requiring serious commitment and holistic personal growth. Analysis of the document analysis results indicates that Taekwondo practitioners experience personal growth and development through their training. These findings align with those of Lee (2022), who observed that youth involvement in Taekwondo can enhance social–emotional skills and facilitate the application of these skills in various aspects of their lives. This is particularly effective when the principles of the SBYD framework are integrated into the context of TKD (e.g., afterschool TKD program, school physical education, and traditional TKD). TKD academies emphasizing positive values are considered traditional martial arts. Traditional martial arts are deeply rooted in Taoism and Buddhism, which for centuries have guided warriors like the Hwarang in Korea to train and live as true warriors (Cook, 2001). The doctrine of Hwarang-do, the way of flowering manhood, is governed by the five codes of human conduct (Sesok-ogye) handed down by a Buddhist monk, emphasizing nine moral virtues: humility, courtesy, wisdom, justice, trust, goodness, virtue, loyalty, and courage (Cook, 2001). Today, traditional martial arts continue to stress ethical and moral guidance, focusing on self-improvement, cultivation, enlightenment, and realization (Cook, 2001; Cynarski and Lee-Barron, 2014). United Nations Education, Scientific and Cultural Organization’s (2019) report supports this, highlighting the use of martial arts as an educational tool to teach essential values and life skills necessary for cultivating a peaceful and nonviolent culture, such as self-respect, respect for others, self-discipline, fair play, resilience, and cultural diversity. Although previous research (e.g., Lee, 2022; Cook, 2001; United Nations Education, Scientific and Cultural Organization, 2019) and the present study demonstrate a link between TKD practice and positive youth developmental outcomes, there is still a need for further examination in the literature on SBYD and youth martial arts programming to understand “what works” in producing these outcomes.

### 5.2 Goal setting

The findings of this study highlight the importance of goal setting in the journey to achieving a TKD black belt, aligning with principles in SBYD. Goal setting, a key component of the SBYD framework, is crucial for fostering positive developmental outcomes among youth (Bean et al., 2018; Weiss et al., 2014). Research in SBYD highlights that effective goal setting involves clear, measurable, and achievable objectives (see SUPER program, Danish et al., 2005). In TKD, the belt ranking system provides a structured mechanism for setting both short-term and long-term goals, which are instrumental in maintaining motivation and promoting self-improvement among practitioners. The progression through colored belts serves as a tangible marker of progress, with each belt representing a milestone and offering a series of short-term objectives culminating in the ultimate goal of achieving a black belt. The incremental nature of earning tips or stripes for each skill within a belt rank exemplifies the principles of goal setting addressed in the SBYD literature (Danish et al., 2002; Petitpas et al., 2005; Weiss et al., 2014). For example, Hellison’s Teaching Personal and Social Responsibility Model (Hellison, 2011) emphasizes five key goals: (1) respect for the rights and feelings of others, (2) effort and cooperation, (3) self-direction, (4) helping others and leadership, and (5) transfer of learning outside the gym. These goals are structured into five levels (e.g., Level One: Respect, Level Two: Effort, and Level Five: Transfer) and achieved by youth learners as they demonstrate appropriate behaviors corresponding to each level.

### 5.3 The life skills integration in TKD curriculum

The integration of black belt life skills within the TKD curriculum is a significant finding of this study. The integration further enriches the goal setting process. Each belt level includes specific black belt life skills such as respect, focus, integrity, confidence, cooperation, and perseverance, which are expected to internalize and demonstrate. The explicit inclusion of black belt life skills at each belt level in TKD curricula supports the assertion by Gould and Carson (2008) and Hellison (2011) that effective SBYD programs must intentionally teach and reinforce these skills. SBYD researchers differentiate a youth sport program, which emphasizes the development of physical and athletic skills, from a sport based youth development program, which utilizes sport as a means to simultaneously teach both physical and life skills and employs strategies to foster the transfer of these life skills to other areas of life (Petitpas et al., 2005). Therefore, intentionally teaching life skills through sport and physical activity is a key principle of SBYD programs. This dual focus on hard skills (e.g., TKD techniques) and soft skills (e.g., life skills) in TKD reinforces the holistic approach to positive youth developmental outcomes, which develop well-rounded individuals. This finding aligns with Lee’s (2022) assertion that the yin-yang philosophy is a core value in the traditional TKD curriculum, which focuses on the integration of physical and mental skills to promote the connection between body and mind as a whole person.

Our research findings revealed that the four TKD academies included black belt life skills in both the instructor manual and the curriculum manual. These resources help instructors understand and integrate these skills into specific TKD lessons. Additionally, Taekwondoists were provided with a black belt logbook and belt promotion test forms, which outline specific black belt life skills and examples of expected behaviors. This approach allows Taekwondoists to become aware of these skills and evaluate their behavior. It aligns with Hemphill et al. (2019), who highlighted the effectiveness of using student handbooks—referred to as ‘passport to success’ in their study—to help youth grasp life skills as program goals and guide appropriate behavior.

### 5.4 Implications

This study provides insights into how TKD, as a martial art, can serve as an effective context for SBYD. The integration of life skills within the TKD curriculum, the structured goal-setting framework of the belt ranking system, and youth Taekwondoists experience in overcoming challenges and commitment all contribute to positive youth development outcomes through the black belt journey. The research highlights how traditional martial arts philosophies or values such as the five tenets of TKD (Courtesy, Integrity, Perseverance, Self-Control, and Indomitable Spirit), align with life skills education in sport contexts. This finding suggests that traditional practices and values can be integrated into educational and developmental frameworks, enriching the conceptual understanding of SBYD. The study supports a holistic development approach, demonstrating that TKD practice cultivate physical (TKD skills), emotional (manage anxiety and fear), social (respect), and cognitive skills (remembering and focus). This holistic approach challenges a notion that separate physical education and youth sport programs from social–emotional learning, proposing a more integrated model where various aspects of development are interdependent and mutually reinforcing. By focusing on different TKD academies, the research provides insights into how contextual factors, such as the teaching philosophy of instructors and the cultural setting of the academies, influence PYD outcomes. This suggests the importance of context-specific adaptations in SBYD framework. The findings suggest practical guidelines for designing TKD and other martial arts curricula to promote PYD outcomes. Specifically, the integration of life skills into the dojang (training hall), clearly outlined goals for each belt level, and a structured pathway for physical and life skill development are crucial. These elements can serve as a blueprint for instructors to systematically incorporate such strategies into their practices. The study’s results can inform policy-makers and program leaders about the benefits of including martial arts in school curriculums or community programs aimed at PYD. The positive outcomes associated with Taekwondo, such as improved black belt life skills make a strong case for supporting SBYD using martial arts as a means to foster PYD.

### 5.5 Limitations

The study was limited to four TKD academies in the Southeastern region of the United States. While these academies provided valuable insights into the Taekwondo black belt journey, the findings may not be generalizable to all TKD practitioners or other martial arts disciplines, particularly those outside the United States. The reliance on pre-existing documents, such as black belt essays and training logs, while rich in detail, may not capture the full spectrum of experiences and perspectives. These documents were written by participants and may be influenced by the formal requirements of the black belt test, potentially limiting the expression of more nuanced or negative experiences. Moreover, the study did not include direct interviews or observational data, which could have provided deeper insights and triangulated the findings. The study acknowledges the significant role of instructors and the cultural setting of the academies in shaping the experiences and developmental outcomes of the youth Taekwondoists. However, it does not fully explore the variability in teaching styles, instructor qualifications, or cultural differences that may impact the implementation of the SBYD framework. This may limit the understanding of how different teaching approaches and cultural contexts influence the outcomes. Finally, the study primarily focuses on the positive developmental outcomes associated with the black belt journey. This emphasis may overlook potential negative aspects, such as the stress and pressure associated with achieving high ranks, or the potential for reinforcing aggressive behaviors in some contexts. A more balanced examination of both positive and negative outcomes would provide a more comprehensive understanding of the Taekwondo black belt journey.

### 5.6 Future study

Future research could include interviews with participants, instructors, and parents, as well as direct observations of classes and training sessions. This would provide a more nuanced understanding of the experiences and challenges faced by participants and help to triangulate findings from document analysis. In addition to including more qualitative methods, future research should use quantitative methods, such as surveys and psychological assessments, to measure the impact of Taekwondo training on specific developmental outcomes, such as self-esteem, self-control, and social skills. Future research into the role of different teaching styles and instructor qualifications in shaping the developmental outcomes of youth in Taekwondo programs is needed to better understand the effectiveness of traditional versus modern teaching methods, or the impact of instructors’ cultural backgrounds on their teaching approach. Further examination of instructional strategies for life skills teaching used in difference contexts of martial arts could develop insight into in best practices for integrating life skills into martial arts practice. Further research into the application of black belt life skills outside of Taekwondo could enhance our understanding of how these skills are transferred. For example, black belt life skills might be applicable to other sports, the workplace, or academic settings.

## Data Availability

The datasets presented in this article are not readily available because this research dataset is only used for research purposes. Requests to access the datasets should be directed to SL, smlim@inu.ac.kr.
